# ROCK1 knockdown inhibits non-small-cell lung cancer progression by activating the LATS2-JNK signaling pathway

**DOI:** 10.18632/aging.103386

**Published:** 2020-06-17

**Authors:** Ting Xin, Wei Lv, Dongmei Liu, Yongle Jing, Fang Hu

**Affiliations:** 1 Department of Cardiology, Tianjin First Central Hospital, Tianjing 300192, P.R. China

**Keywords:** ROCK1, LATS2, JNK, NSCLC, apoptosis

## Abstract

Rho-associated kinase 1 (ROCK1) regulates tumor metastasis by maintaining cellular cytoskeleton homeostasis. However, the precise role of ROCK1 in non-small-cell lung cancer (NSCLC) apoptosis remains largely unknown. In this study, we examined the function of ROCK1 in NSCLS survival using RNA interference-mediated knockdown. Our results showed that ROCK1 knockdown reduced A549 lung cancer cell viability *in vitro*. It also inhibited A549 cell migration and proliferation. Transfection of ROCK1 siRNA was associated with increased expression of large tumor suppressor kinase 2 (LATS2) and c-Jun N-terminal kinase (JNK). Moreover, ROCK1 knockdown-induced A549 cell apoptosis and inhibition of proliferation were suppressed by LATS2 knockdown or JNK inactivation, suggesting that ROCK1 deficiency triggers NSCLC apoptosis in a LATS2-JNK pathway-dependent manner. Functional analysis further demonstrated that ROCK1 knockdown dysregulated mitochondrial dynamics and inhibited mitochondrial biogenesis. This effect too was reversed by LATS2 knockdown or JNK inactivation. We have thus identified a potential pathway by which ROCK1 downregulation triggers apoptosis in NSCLC by inducing LATS2-JNK-dependent mitochondrial damage.

## INTRODUCTION

Non-small-cell lung cancer (NSCLC) is a main cause of cancer death worldwide and accounts for approximately 80–85% of all lung cancer cases [1]. Recent advances in diagnosis and treatment have improved the survival of patients with early stage NSCLC [2]. Although a growing number of studies have examined the molecular mechanisms of NSCLC carcinogenesis, they remain largely unknown. In addition, the mild symptoms and clinical signs that characterize early-stage NSCLC can preclude early detection, and NSCLC progresses rapidly. Most patients are diagnosed at advanced stages, resulting in poor prognosis and low survival rates [3]. Importantly, some NSCLC subtypes are highly resistant to radiotherapy/chemotherapy and are therefore treated mainly by surgery [4]. Additional studies are needed to improve understanding of the pathogenesis and molecular mechanisms of NSCLC and to identify potential treatment targets to reduce NSCLC cell survival, invasion, and proliferation.

Rho-associated kinase 1 (ROCK1) was originally described as a downstream effector of the small GTPase RhoA. As a serine/threonine kinase, ROCK1 plays an important role in sustaining cellular cytoskeleton turnover by promoting the formation F-actin [5, 6]. Interestingly, F-actin synthesis and degradation are closely associated with the migration and mobilization of various types of tumors, including liver cancer [7], endometrial cancer [8], prostate cancer [9], colorectal cancer [10], breast cancer [11] and lung cancer [12]. This suggests that ROCK1 might also affect lung cancer development and progression [13]. Other studies have demonstrated that ROCK1 enhances mitochondrial function [14], augments cellular energy metabolism [15], and promotes cell cycle transition [16]. These findings indicate that ROCK1 may modulate lung cancer bioenergetics and proliferation. Although a recent study demonstrated that ROCK1 is involved in lung cancer migration/invasion [12], the mechanisms by which ROCK1 impacts lung cancer survival are not fully understood.

At the molecular level, large tumor suppressor kinase 2 (LATS2) has been identified as a novel pro-apoptotic factor. Together with macrophage stimulating 1 (Mst1), LATS2 interacts with BCL-xL to inhibit the activity of mitochondria-related anti-apoptotic proteins such as Bcl2 or c-IAP1 [17, 18], leading to an imbalance between Bax and Bcl2. Under physiological conditions, Bcl2 interacts with Bax to block Bax-mediated mitochondrial damage [19, 20]. Once inactivated by LATS2, Bax is separated from Bcl2 and inserted into the outer mitochondrial membrane [21], leading to mitochondrial damage. In addition, LATS2 acts as an agonist of the JNK pathway by promoting post-transcriptional modification of JNK [22, 23]. Once phosphorylated, JNK translocates into the nucleus, where it induces the transcription of pro-apoptotic proteins such as Bad and Bax and ultimately leads to apoptosis. The causal relationship between ROCK1 upregulation and LATS2 inhibition was first described in the context of type-2 diabetes [24, 25]. Whether ROCK1 promotes cancer cell viability by inhibiting LATS2 and subsequently blocking Bax- or JNK-induced mitochondrial apoptosis in NSCLC remains to be determined. The aim of this study was to characterize the role of ROCK1 in lung cancer survival with a focus on the LATS2-JNK signaling pathway. To that end, A549 cells were transfected with siRNA against ROCK1 in a loss-of-function assay, and LATS2 expression and JNK transcription were measured. To determine whether LATS2 and JNK acted as downstream effectors of ROCK1, ROCK1-knockdown A549 cells were then treated with LATS2 siRNA and a JNK inhibitor.

## RESULTS

### ROCK1 knockdown reduces A549 cell viability and induces cell apoptosis

**Figure 1:**
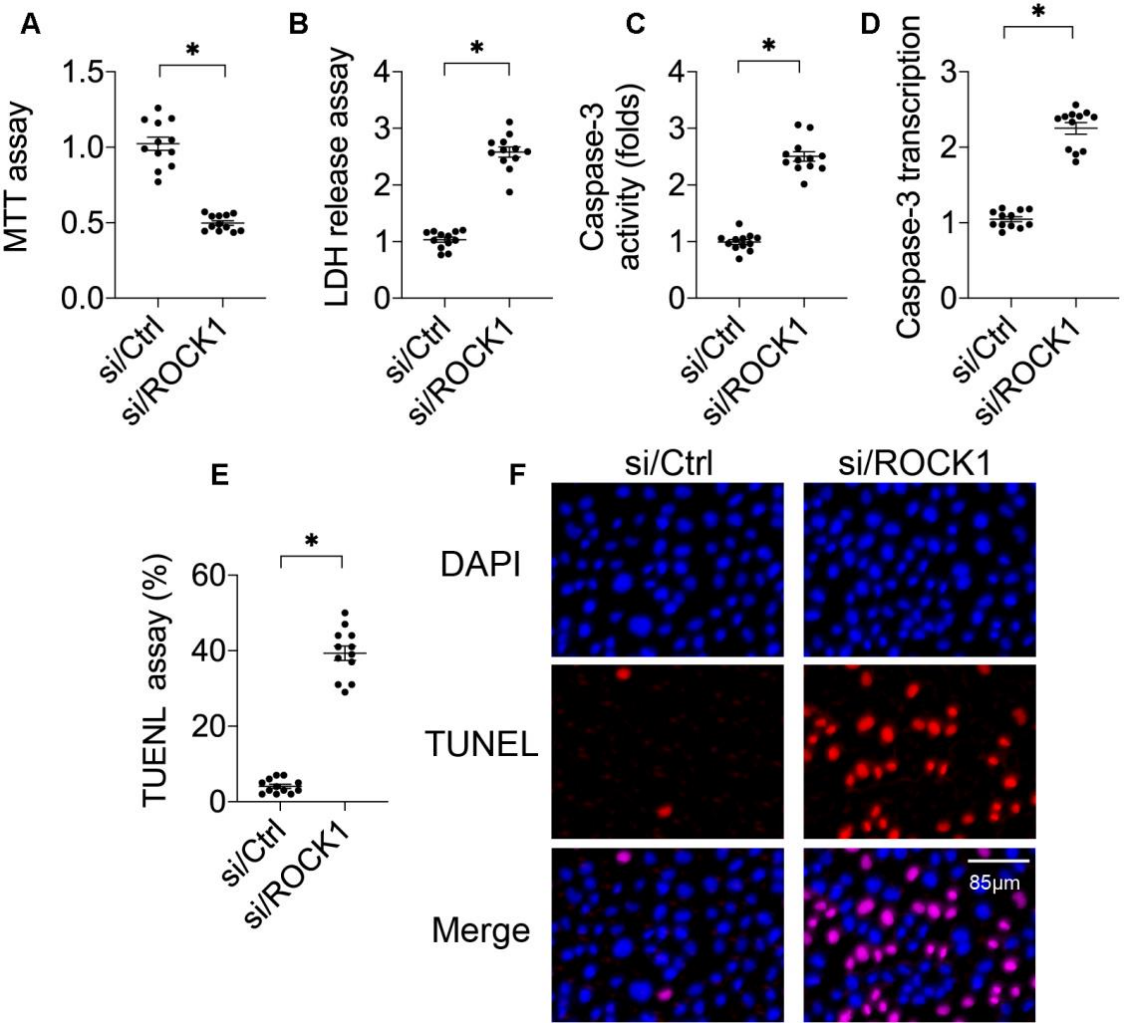
**ROCK1 regulates A549 cell viability.** (**A**) MTT assay for A549 cells. A549 cells were transfected with siRNA against ROCK1 (si/ROCK1) or control siRNA (si/Ctrl). (**B**) An LDH release assay was used to measure LDH levels in the medium of A549 cells transfected with siRNA against ROCK1 (si/ROCK1) or control siRNA (si/Ctrl). (**C**) ELISA was used to analyze Caspase-3 activity in response to si/ROCK1 or si/Ctrl transfection. (**D**) A qPCR assay was used to measure Caspase-3 transcription. (**E**, **F**) TUNEL staining was used to measure numbers of apoptotic cells in response to si/ROCK1 or si/Ctrl transfection. *p<0.05.

A549 cells were transfected with siRNA against ROCK1 (si/ROCK1) to observe the effects of ROCK1 in NSCLC development and progression. Compared to the control, si/ROCK1 transfection reduced cell viability in an MTT assay (Figure 1A), indicating that ROCK1 was necessary for cancer survival. An LDH release assay was then performed to confirm this result. Under normal conditions, LDH is contained in the cytoplasm [26, 27]; during cellular membrane breakdown indicative of decreased cell viability, LDH is released into the culture medium [28, 29]. Compared to the control group, si/ROCK1 transfection increased LDH levels in the culture medium (Figure 1B), suggesting that ROCK1 deficiency is associated with cell membrane breakdown. Next, we examined the effects of ROCK1 knockdown on cell apoptosis by measuring the activity and transcription of caspase-3 protein, a key apoptosis promoter that triggers cell membrane breakdown [30, 31]. As shown in Figure 1C, 1D, compared to the control group, caspase-3 activity and transcription were dysregulated in response to si/ROCK1 transfection. Finally, TUNEL staining was used to quantify numbers of apoptotic A549 cells. As shown in Figure 1E, 1F, compared to the control group, the number of TUNEL-positive cells increased after exposure to si/ROCK1, confirming that inhibition of ROCK1 upregulated apoptosis in A549 cells. Together, these results indicate that ROCK1 knockdown activates apoptosis and thus reduces NSCLC cell viability in *in vitro*.

### ROCK1 knockdown reduces NSCLC migration and proliferation

**Figure 2:**
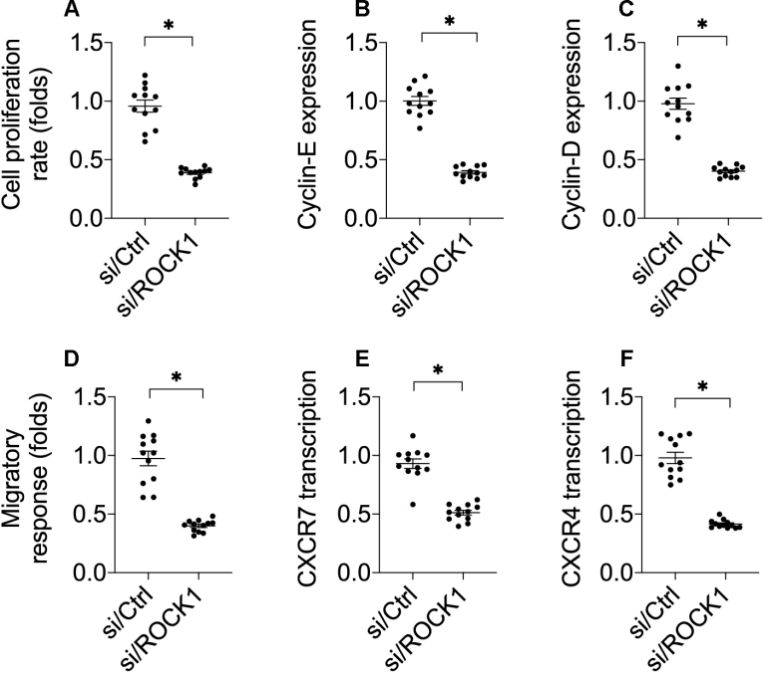
**ROCK1 knockdown decreases cell migration and proliferation.** (**A**) A CCK-8 assay was used to quantify proliferation in A549 cells transfected with siRNA against ROCK1 (si/ROCK1) or control siRNA (si/Ctrl). (**B**, **C**) A qPCR assay was used to analyze Cyclin-D and Cyclin-E transcription. (**D**) Transwell assay for A549 cells. Numbers of migrated cells were quantified after si/ROCK1 or si/Ctrl transfection. (**E**, **F**) A qPCR assay was used to analyze CXCR-4 and CXCCR-7 transcription. *p<0.05.

Cancer cell invasion and proliferation are crucial to cancer progression; we therefore examined whether ROCK1 also regulated A549 cell proliferation and mobilization [32]. As shown in Figure 2A, a CCK-8 assay demonstrated that cell proliferation was impaired by si/ROCK1. Accordingly, cyclin-D and cyclin-E transcription were also downregulated in A549 cells after transfection of si/ROCK1 (Figure 2B, 2C). These results indicate that ROCK1 knockdown inhibits NSCLC proliferation.

Next, we examined the effects of ROCK1 on A549 cell migration and invasion *in vitro*. In a transwell assay, A549 cell migration was drastically reduced after si/ROCK1 transfection (Figure 2D). At the molecular level, CXCR-4 and CXCR-7 have been identified as key promoters of cancer adhesion and migration. Interestingly, transfection of si/ROCK1 decreased their transcription in A549 cells (Figure 2E, 2F). These results confirmed that inhibition of ROCK1 impairs NSCLC proliferation and migration.

### ROCK1 knockdown activates LATS2-JNK pathways

**Figure 3:**
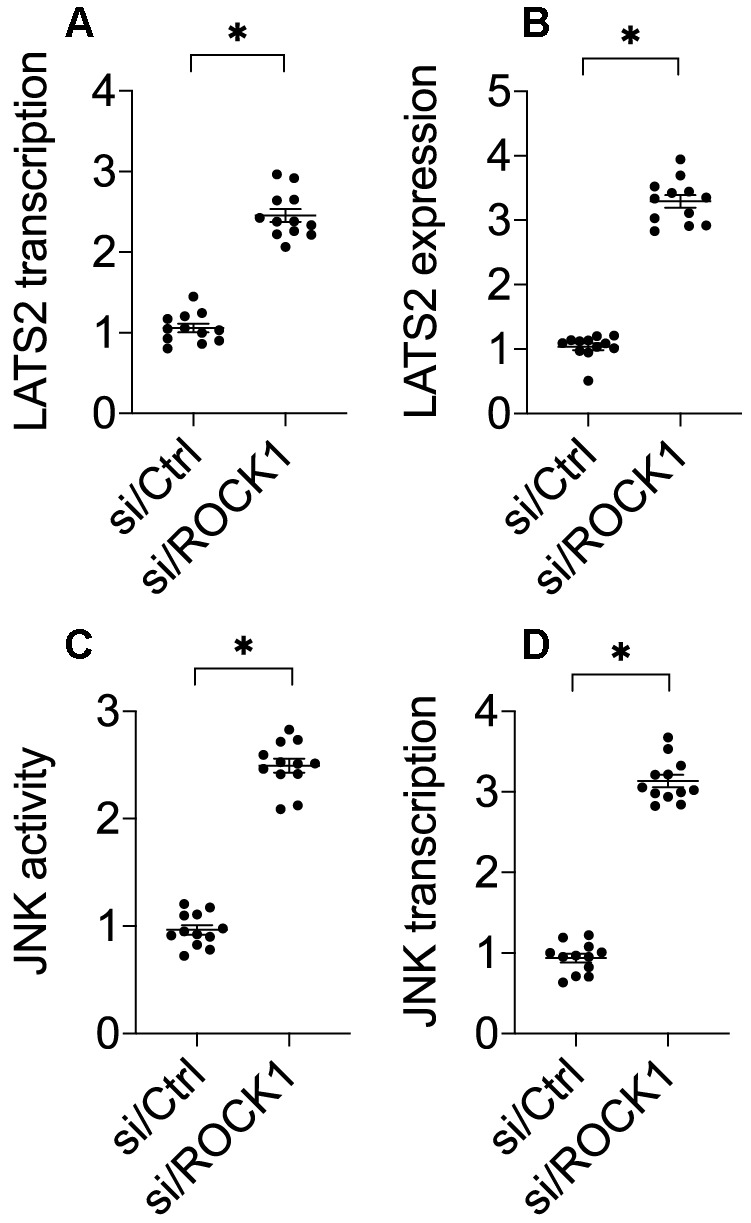
**The LATS2-JNK pathway is activated after ROCK1 knockdown.** (**A**) A qPCR assay was used to analyze LATS2 transcription. (**B**) Western blots were used to detect LATS2 protein levels in response to si/ROCK1 transfection. (**C**) An ELISA was used to measure JNK activity in response to siRNA-mediated ROCK1 knockdown. (**D**) A qPCR assay was used to analyze JNK transcription after si/ROCK1 transfection. *p<0.05.

Previous studies have demonstrated that ROCK1 activation is associated with LAST2 suppression [33]. Interestingly, recent experiments have identified LATS2 as a novel regulator of cancer survival and invasion [34, 35]. We therefore explored whether ROCK1 knockdown-mediated A549 cell apoptosis was attributable to LATS2 activation. Compared to the control group, si/ROCK1 treatment increased LATS2 RNA (Figure 3A) and protein levels (Figure 3B), suggesting that ROCK1 knockdown promotes LATS2 transcription and translation. Notably, the pro-apoptotic mitochondrial protein JNK functions downstream of LATS2 [36, 37]. Given that JNK activation promotes mitochondrial apoptosis [7, 38], we examined whether ROCK1 knockdown-induced apoptosis was mediated by JNK activation in A549 cells. In an ELISA, si/ROCK1 transfection increased JNK kinase activity (Figure 3C); this increase was accompanied by an upregulation of JNK transcription (Figure 3D). Together, these data indicate that ROCK1 knockdown is associated with an activation of LATS2 and JNK in NSCLC *in vitro*.

### Loss of LATS2 or JNK abolishes the tumor-suppressing effects of ROCK1 knockdown

**Figure 4:**
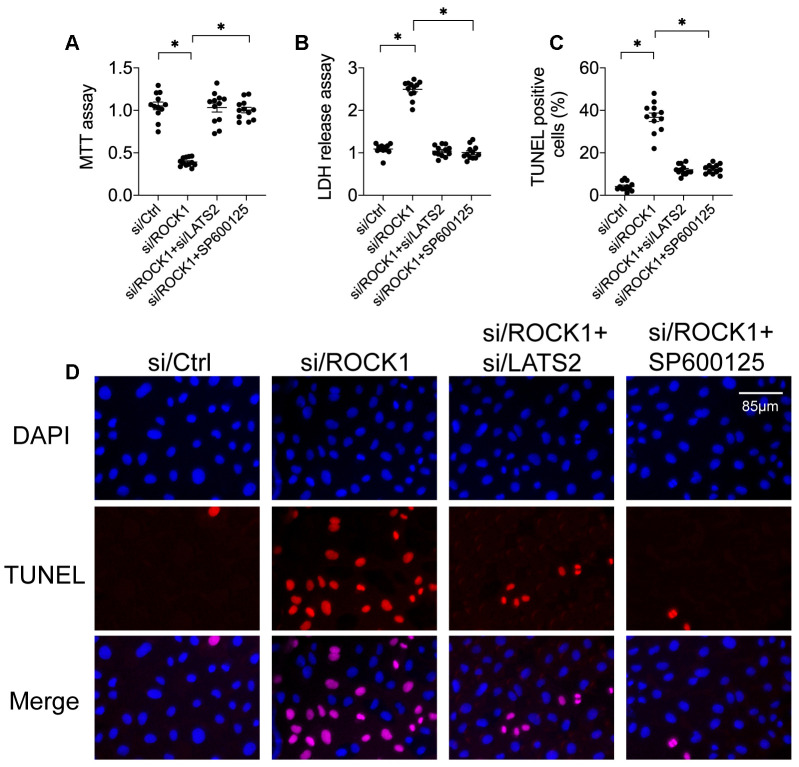
**Inactivation of the LATS2-JNK pathway abolishes the tumor-suppressive effects of ROCK1 knockdown.** (**A**) MTT assay of cell viability. siRNA against LATS2 (si/LATS2) and SP600125 were used inhibit LATS2 upregulation and JNK activation, respectively. (**B**) An LDH release assay was used to measure LDH levels in the medium. (**C**, **D**) TUNEL staining was used to quantify numbers of apoptotic cells after si/LATS2 transfection and SP600125 administration. *p<0.05.

To further explore potential causal relationships between ROCK1 expression and the LATS2-JNK pathway in A549 cells [39], siRNA against LATS2 (si/LATS2) was transfected before si/ROCK1 transfection to interrupt ROCK1 deficiency-induced LATS2 activation. An MTT assay demonstrated that si/ROCK1 treatment reduced cell viability in A549 cells compared to the control group, while co-transfection of si/LATS2 restored cell viability almost to control levels (Figure 4A). A similar effect was observed in the LDH release assay; although si/ROCK1 transfection increased LDH levels in the medium, si/LATS2 co-transfection reversed this increase (Figure 4B). In agreement with these results, si/ROCK1 transfection increased, while co-transfection of si/LATS2 reduced, the number of TUNEL-stained A549 cells (Figure 4C, 4D). These data indicate that ROCK1 knockdown-induced A549 cell apoptosis could be abolished by LATS2 inhibition.

To determine whether JNK inactivation also blunted ROCK1 knockdown-induced damage, A549 cells were incubated with the JNK pathway inhibitor SP600125 before si/ROCK1 transfection. As was observed after transfection of si/LATS2, SP600125-induced inactivation of the JNK pathway also restored A549 cell viability in the MTT assay (Figure 4A). Moreover, inhibition of JNK also attenuated ROCK1 knockdown-induced increases in medium LDH levels (Figure 4B). In addition, SP600125 also inhibited ROCK1 knockdown-induced increases in A549 cell apoptosis as indicated by TUNEL staining (Figure 4C, 4D). These results suggest that inhibition of either LATS2 or JNK impairs the tumor-suppressive effects of ROCK1 knockdown and promotes A549 cell survival.

### LATS2 knockdown or JNK inactivation attenuate ROCK1 knockdown-induced mitochondrial apoptosis

**Figure 5:**
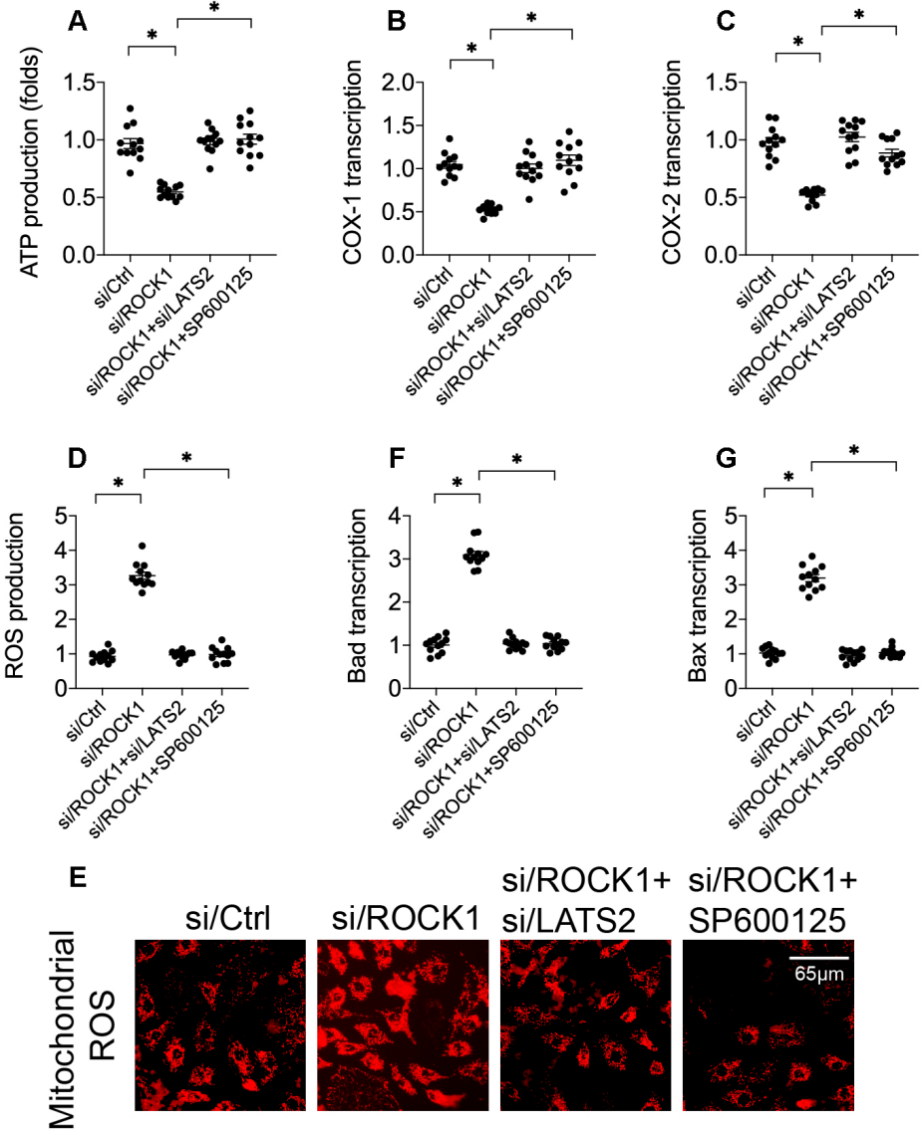
**ROCK1 deficiency promotes mitochondrial apoptosis by activating the LATS2-JNK pathway.** (**A**) ATP production was measured in A549 cells after transfection of siRNA against LATS2 (si/LATS2) and SP600125 administration that inhibited LATS2 upregulation and JNK activation, respectively. (**B**, **C**) qPCR was used to analyze COX-1 and COX-2 transcription. (**D**, **E**) Immunofluorescence was used to measure generation of ROS in A549 cells. (**F**–**G**) A qPCR assay was used to detect changes in Bax and Bad levels in A549 cells. *p<0.05.

To investigate the molecular mechanisms underlying LATS2-mediated A549 cell death, we next examined mitochondrial apoptosis, as mitochondria are a potential target of the LATS2-JNK pathway [40, 41]. First, we evaluated mitochondrial function by analyzing cytoplasm ATP levels. Compared to the control group, ATP production was reduced in A549 cells transfected with si/ROCK1 (Figure 5A). Interestingly, this effect was reversed by si/LATS2 or SP60125, suggesting that ROCK1 may sustain mitochondrial metabolism by inhibiting the LATS2-JNK pathway. To further confirm this finding, levels of the mitochondrial respiration-related proteins cyclooxygenase-1/2 (COX-1/2) were measured. As shown in Figure 5B, 5C, compared to the control group, COX-1 and COX-2 levels were significantly downregulated in response to si/ROCK1 transfection. However, LATS2 knockdown or JNKinhibition reversed this effect, confirming that ROCK1 knockdown-induced disruption of mitochondrial metabolism is a result of LATS2/JNK pathway activation.

In addition to decreased ATP production, we found that generation of ROS increased after si/ROCK1 transfection (Figure 5D, 5E), suggesting that ROCK1 deficiency increases oxidative stress injuries in A549 cells. However, inhibition of the LATS2/JNK pathway attenuated this accumulation of ROS in A549 cells (Figure 5D, 5E). Transcription of mitochondrial pro-apoptotic proteins Bax and Bad was similarly affected by si/ROCK1 transfection and LATS2/JNK pathway inhibition. Compared to the control group, Bax and Bad transcription were upregulated by si/ROCK1 transfection and restored by inactivation of the LATS2/JNK pathway in A549 cells (Figure 5F, 5G). Taken together, our results indicate that ROCK1 knockdown triggers mitochondrial apoptosis, and that blockade of the LATS2-JNK pathway inhibited this effect.

### Mitochondrial dynamics and mitochondrial biogenesis are controlled by the ROCK1-LATS2-JNK pathway

**Figure 6:**
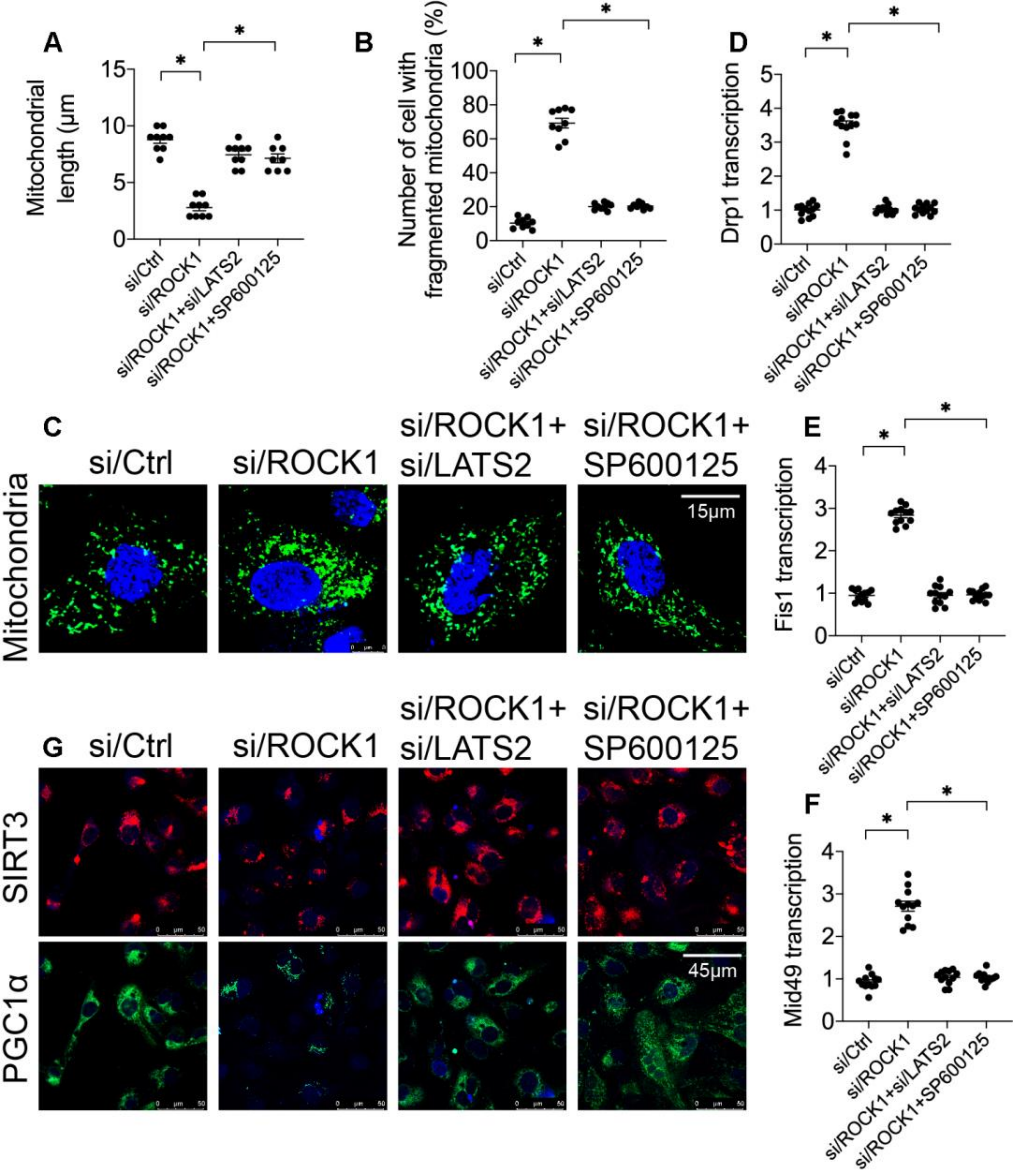
**The ROCK1-LATS2-JNK pathway affects mitochondrial dynamics and mitochondrial biogenesis in A549 cells.** (**A**–**C**) Immunofluorescence was used to observe mitochondrial morphology in A549 cells. siRNA against LATS2 (si/LATS2) and SP600125 were used inhibit LATS2 upregulation and JNK activation, respectively. Mitochondrial length and number of cells with fragmented mitochondria were recorded. (**D**–**F**) A qPCR assay was used to analyze Drp1, Fis1, and Mid49 transcription in A549 cells in response to ROCK1 knockdown, LATS2 knockdown, and JNK inhibition. (**G**) Double immunofluorescence was used to observe alterations in Sirt3 and PGC1α levels; relative immunofluorescence intensities were evaluated. *p<0.05.

Recent studies indicate that changes in mitochondrial dynamics and inhibition of mitochondrial biogenesis occur early in the mitochondrial apoptosis induction process [29, 42]. To further understand the regulatory mechanism by which the ROCK1-LATS2-JNK pathway induced mitochondrial apoptosis, mitochondrial dynamics and biogenesis were analyzed. First, immunofluorescence was used to identify alterations in mitochondrial morphology in A549 cells after transfection of si/ROCK1 together with either si/LATS2 or SP600125 treatment. Compared to the control group, si/ROCK1 transfection increased numbers of fragmented mitochondria, the length of which was reduced to ~2.7 μm (Figure 6A–6C). The addition of either si/LATS2 or SP600125 reduced numbers of fragmented mitochondria and increased average mitochondrial length to ~9.7 μm (Figure 6A–6C). These results indicate that ROCK1 knockdown altered mitochondrial dynamics in a LATS2/JNK pathway-dependent manner. To further examine this possibility, we examined transcription of the mitochondrial dynamics-associated markers Drp1, Fis1, and Mid49. Compared to the control group, Drp1, Fis1, and Mid49 transcript levels increased after transfection with si/ROCK1; this effect was abrogated by si/LATS2 or SP600125 (Figure 6D–6F).

To understand the influence of the ROCK1/LATS2/JNK pathway on mitochondrial biogenesis, alterations in PGC1α and Sirt3 expression were measured using double-immunofluorescence in A549 cells [43]. As shown in Figure 6G, PGC1α and Sirt3 expression were downregulated by si/ROCK1 in A549 cells, and loss of LATS2 or inactivation of JNK reversed this effect; thus, ROCK1 knockdown inhibited mitochondrial biogenesis via the LATS2/JNK pathway. Together, these results suggest that ROCK1/LATS2/JNK pathway activity promotes NSCLC apoptosis by inducing dysregulation of mitochondrial dynamics and inhibiting mitochondrial biogenesis.

### DISCUSSION

Methods for detecting, diagnosing, and treating NSCLC have improved greatly in recent decades. However, the molecular mechanisms underlying the development, progression, and metastasis of NSCLC remain largely unknown, and additional basic research and clinical trials are needed [44]. NSCLC development and progression are affected by both genetic and environmental factors and are driven by activation of multiple oncogenes and inactivation of tumor suppressor genes [45, 46]. Furthermore, prognoses are generally poor for patients with lung cancer, especially those with advanced stage disease at the time of diagnosis. Genetic factors, including mutations, proto-oncogene mismatches, and CpG island methylation modifications in anti-oncogene promoter regions, are associated with NSCLC pathogenesis [47]. In the present study, we identified ROCK1 as a novel promoter of NSCLC viability. Mechanistically, ROCK1 knockdown upregulated LATS2 and thus activated the JNK pathway, which promoted mitochondrial damage by increasing mitochondrial apoptosis, dysregulating mitochondrial dynamics, and inhibiting mitochondrial biogenesis. Our findings suggest that inhibition of ROCK1 and/or activation of the LATS2/JNK pathway might be a promising approach for suppressing NSCLC survival via mitochondrial injury.

The tumor-suppressive effects of ROCK1 knockdown have been observed in many different types of cancers. For example, esophageal squamous cell carcinoma invasion and migration are attenuated by ROCK1 deletion *in vitro* [48]. In addition, TGFβ-induced epithelial-mesenchymal transition in NSCLC is also regulated by ROCK1 in a miR-335-5p-dependent manner [49]. In addition, increased cytoplasmic ROCK1 levels are necessary for maintaining proliferation and survival in human myeloma cells [50]. In prostate cancer, increased ROCK1 expression has been defined as an early biomarker for poor prognosis due to its association with genetic instability in tumor cells [51]. Similarly, we found here that moderate cytoplasmic ROCK1 expression was vital for NSCLC survival. RNA interference-mediated ROCK1 knockdown drastically increased numbers of apoptotic cells *in vitro.* Several compounds have been identified that inhibit ROCK1 activity and might therefore prove effective for treating NSCLC. For example, histamine [52], fibroblast-derived hepatocyte growth factor [53], triptolide [54], and melatonin [55, 56] have been used to inhibit ROCK1 activation. However, additional clinical studies and pre-clinical experiments are needed to support the use of these and other compounds as clinically useful targeted therapeutic agents in NSCLC patients.

The effects of ROCK1 inhibition on NSCLC apoptosis are dependent on increased LATS2 expression and JNK activation that induce mitochondrial damage. In addition to controlling cellular energy metabolism, mitochondria are also important regulators of redox balance, calcium homeostasis, protein oxidation, and cell death [57–59]. Indeed, mitochondria are the key target of several anti-cancer drugs, such as fluorouracil [60], silibinin [61], resveratrol [62], sorafenib [63], and matrine [64]. Here, we report that mitochondrial function and morphology were controlled by the LATS2-JNK pathway. Increased LATS2 expression may increase transcription of mitochondrial dynamics-related proteins, such as Drp1, Fis1, and Mid49, leading to mitochondrial fragmentation and reduced mitochondrial potential. Increased LATS2 levels were also associated with decreases in the levels of transcription of factors related to mitochondrial biogenesis, suggesting that LATS2 activation might interrupt mitochondrial self-renewal. Taken together, these results suggest that the tumor-suppressive effects of the LATS2-JNK pathway are likely due to both the induction of mitochondrial fragmentation and disruption of mitochondrial turnover. To our knowledge, this is the first study to describe this relationship between LATS2-JNK pathway activation and mitochondrial damage in NSCLS.

Overall, our results demonstrated that non-small-cell lung cancer viability is regulated by ROCK1 and the LATS2-JNK pathway. Mechanistically, ROCK1 knockdown activated the LATS2-JNK pathway, which in turn dysregulated mitochondrial dynamics and inhibited mitochondrial biogenesis, possibly at the post-transcriptional level. These finding suggest that ROCK1 and LATS2-JNK may be potential targets for NSCLC treatments.

## MATERIALS AND METHODS

### Cell culture and siRNA transfection

The A549 lung cancer cell line was purchased from the Korean Cell Line Bank. RPMI-1640 medium containing 10% fetal bovine serum, 1% penicillin/streptomycin, and 2-mercaptoethanol was used to culture A549 cells in a culture flask at 37°C in a 5% CO_2_ atmosphere [65]. A549 cells at passage 5-8 were transiently transfected with scramble (Scr) siRNA (Invitrogen, #12935110), ROCK1 siRNA, and LATS2 siRNA as indicated. All siRNAs were predesigned and purchased from Thermo Fisher Scientific. Two days after transfection, cells were cultured in serum-free media for 21 hr and stimulated with Ang II (100 nM) for 3 hr. Western blots or qPCR were used to verify transfection and knockdown efficiency [66].

### Terminal deoxynucleotidyl transferase nick-end-labeling (TUNEL)

We used a TUNEL kit (11684817910, Roche, Indianapolis, IN, USA) as described by the manufacturer [67, 68]. A549 cell samples were dewaxed and rehydrated. Endogenous peroxidase activity was blocked using 3% hydrogen peroxide for 5 minutes. The samples were then washed with phosphate-buffered saline (PBS) at room temperature and incubated in TUNEL Reaction Mixture followed by converter-POD solution at 37°C. Next, the slides were incubated with diaminobenzidine (DAB) and stained with hematoxylin [69]. Samples were dehydrated using graded ethanol, vitrified with dimethylbenzene, and deposited in neutral resins. Finally, the samples were observed under a microscope.

### TMRE staining

After transfection with siRNA, A549 cells were incubated with 50 pM tetramethylrhodamine ethyl ester (TMRE) for 10 min [70], washed twice with PBS 1x, harvested, centrifuged (1600×g for 4 min at 4°C), and resuspended (about 1×10^6^ cells/mL) in PBS for immunofluorescence analysis. Carbonilcyanide p-triflouromethoxyphenylhydrazone (FCCP), an uncoupling agent that completely depolarizes the outer mitochondrial membrane [71, 72], was used as a positive control. FCCP was added to cell cultures at a final concentration of 20 µM for 20 minutes immediately preceding incubation with TMRE. At least three independent experiments were performed.

### ROS assessment

Cells were grown overnight and then diluted in fresh media to an OD (λ= 660 nm) of 0.2. Then, samples were washed twice in PBS and incubated with 1 mL of 2.5 µg/mL dihydroethidium (DHE) in phosphate buffered saline (PBS) for 15 minutes in the dark [73]. Then, cells were washed with 1 mL PBS and analyzed by immunofluorescence [74]. At least three independent experiments were performed.

### RNA isolation, reverse transcription, and qPCR

Total RNA was isolated from cells using the GeneJet RNA Purification Kit (Thermo Scientific, K0732), and 0.5 μg of total RNA was reverse transcribed to generate cDNA using the iScript cDNA Synthesis Kit (Bio-Rad, 1708891) according to manufacturer’s instructions [75, 76]. qPCR was performed using iTaq Universal SYBR Green supermix (Bio-Rad, 1725121) on Bio-Rad CFX-384 or CFX-96 real-time PCR Systems. Actin was used for normalization [77].

### Proliferation and viability assay

A549 cells were transfected with siRNA for 3 days followed by starvation for 24 hours [78]. Afterwards, all cells were re-plated into 96-well plates at a density of 1×10^3^ cells per well and maintained in 1% FBS-containing DMEM/F12 medium for 24 hours. Then, cell number was assessed using a cell counting kit (CCK8, MedChem Express) according to manufacturer’s instructions [79].

### Scratch-wound and transwell assay

A549 cell migration was assessed by scratch-wound and transwell assays as described previously. Briefly, confluent A549 cells were transfected with siRNAs [80, 81]. Three days later, cells were subjected to scratch or re-plated in transwell chambers, and images were taken at indicated time points. Distances between wound edges and numbers of cells that migrated between the transwell chambers were measured using ZEN analysis software [82].

### Immunostaining

Samples were harvested, immediately fixed with 4% paraformaldehyde for 30 min, and blocked in a 5% solution of normal serum (same type in which secondary antibody was raised) containing 0.1% Triton-X for 1 hr at 37°C. Primary antibody incubations were performed overnight at 4°C [83]. After washing, highly cross-absorbed Alexa 488, Alexa 546, or Alexa 647-conjugated secondary antibodies (Invitrogen) were added at a dilution of 1:200 for 2 hr at 37°C. Control experiments were performed by omitting the primary antibody. Slides were covered with Vectashield containing DAPI (Vector Laboratories) and viewed under a LSM 710 (ZEISS) or Axioscan Z1 (ZEISS) microscope [84, 85].

### Statistical analysis

We conducted a Shapiro-Wilk normality test for the samples. Between-groups independent t-tests, one-way analysis of variance (ANOVA), and two-way ANOVA were used if the variables satisfied the normality assumption. Otherwise, Mann-Whitney U tests, Kruskal-Wallis one-way ANOVAs, and Friedman two-way ANOVAs were used. Bonferroni corrections were used for post-hoc multiple comparisons in ANOVA. Statistical analyses were performed with SAS (version 9.4, SAS Institute, Inc., Cary, NC), Prism 6 (GraphPad Software, La Jolla, CA), or InStat (GraphPad Software) [86].
